# Implementation and Feasibility of a Co-designed Intervention to Promote Healthy Eating: Designed by Children for Children

**DOI:** 10.1007/s10935-026-00913-x

**Published:** 2026-04-10

**Authors:** Urte Klink, Hannah Jilani

**Affiliations:** Department for Health Services Research, Institute for Public Health and Nursing Research, University Bremen, Universitaetsallee 1c, D-28359 Bremen, Germany

**Keywords:** Child, Adolescent, Community-based participatory research, Empowerment, Health behaviour, Diet

## Abstract

The development of healthy dietary behaviours during childhood is crucial for long-term health, and schools represent a key setting for health promotion. Actively involving children in health-related projects offers a meaningful opportunity to encourage and reinforce healthy behaviours. Therefore, this formative pilot study primarily aimed to explore the feasibility, acceptability, and engagement potential of a participatory, school-based dietary intervention involving preadolescent students. During the 2023/24 school year, a participatory research project including a formative pilot study was conducted at a school in Bremen, Germany, aiming to involve children in the co-design, implementation, and formal evaluation of a school-based dietary intervention. Sixth-grade students (11–12 years old) collaborated with researchers over several months to design an intervention for their 5th -grade peers (10–12 years old). The intervention included the free distribution of self-prepared healthy snacks and the display of educational posters. Implementation experiences and participant responses were explored using a child-friendly food neophobia questionnaire, measuring children’s reluctance to try unfamiliar foods, alongside narrative methods. Participation in this pilot study was perceived as highly positive by 6th -grade students, who showed increased confidence, self-esteem, and interest as well as skills in nutrition and cooking/baking. The collaborative process fostered strong relationships between students and researchers and supported sutdents’ sense of empowerment. Although 5th -grade students generally enjoyed the free snacks, their food neophobia scores showed no significant change, consistent with the formative nature of the study. Overall, the findings highlight the feasibility and acceptability of participatory approaches to school-based nutrition interventions and underscore their potential to promote engagement and empowerment among children. Our results provide valuable insights into participatory development and implementation processes to inform future school-based health promotion efforts.

## Introduction

Childhood and adolescence are periods of rapid growth, development, and transition in which health-related behaviours begin to take shape and can have implications across the life course. During these stages, dietary patterns are established that may persist into adulthood and are associated with later health outcomes (Ogata & Hayes, 2014; Wang & Stewart, 2013).

Schools are a crucial place for the healthy development of young people. They are ideal settings for health promotion measures as virtually all children and adolescents spend most of their time there, making them easily reachable (Forneris et al., 2010). Accordingly, health interventions in schools can represent key opportunities to support the development of long-lasting positive health behaviours, including healthy dietary behaviours (O’Brien et al., 2021; Wang & Stewart, 2013). Researchers and stakeholders agree that school-level dietary interventions are critical for promoting children’s health (Heseker & Beer, 2004; WHO, 2020). Such interventions have the potential to create supportive environments that empower children to make healthy dietary choices and ultimately mitigate the long-term health risks linked to overweight and obesity (Heseker & Beer, 2004; Stang, 2010; Wang & Stewart, 2013; WHO, 2020).

Participatory approaches that actively involve the target group in the planning, development, and implementation of dietary and health-related interventions have recently come into focus. These approaches can improve cultural relevance, acceptability among participants, and sustainability in the community (Israel et al., 1998). While adult community members are an important group to engage, it is equally important to conduct participatory research with young people (Frerichs et al., 2016). Their unique perspective can be integrated into strategies and messages to improve the effectiveness of an intervention among its intended participants (Powers & Tiffany, 2006). In addition, it is recognized that youth have an impact on the healthy eating and physical activity behaviours of their peers (Salvy et al., 2012) and that young people may be more inclined to change health behaviours when messages come from individuals closer in age and similar to themselves (Mellanby et al., 2000). Participatory approaches allow young people to make healthier choices while developing various skills, increasing the likelihood of remaining engaged throughout the intervention (Jacquez et al., 2013). They also have the benefit of empowering young people by giving them a sense of ownership and agency in the process (Wong et al., 2010). Several reviews have summarized findings from participatory approaches involving children and adolescents in dietary and other health-related interventions, highlighting their potential to improve young people’s health behaviours while fostering the development of life and leadership skills (Frerichs et al., 2016; Lavelle et al., 2023; Vangeepuram et al., 2020; Yip et al., 2016).

Building on this evidence, there is still a need to better understand how participatory approaches can be applied in real-world school settings to support the development, implementation, and reflection of health-related initiatives (Jilani et al., 2025). While previous studies have demonstrated the benefits of youth participation in promoting engagement, skills, and empowerment (Wong et al., 2010), few have explored how preadolescent students can actively contribute to designing nutrition-related interventions for their peers and how such processes are experienced in everyday school contexts. Additionally, to our knowledge, no comparable interventions have been conducted in Germany or other German-speaking countries (Lavelle et al., 2023; Vangeepuram et al., 2020).

Therefore, this formative pilot study primarily aimed to explore the feasibility, acceptability, and engagement potential of a participatory, school-based dietary intervention co-designed by preadolescent students. An additional exploratory aim was to investigate the reactions to the intervention by the target group, including engagement, acceptance, and responses assessed via a child-friendly food neophobia scale (FNS) (Laureati et al., 2015), without positioning the study as an evaluation of behavioural change. Our formative pilot study provides valuable insights into the feasibility of child-led intervention development and its implementation in a real school context, offering lessons that can inform future school-based health promotion and prevention practice.

## Methods

**Figure 1:**
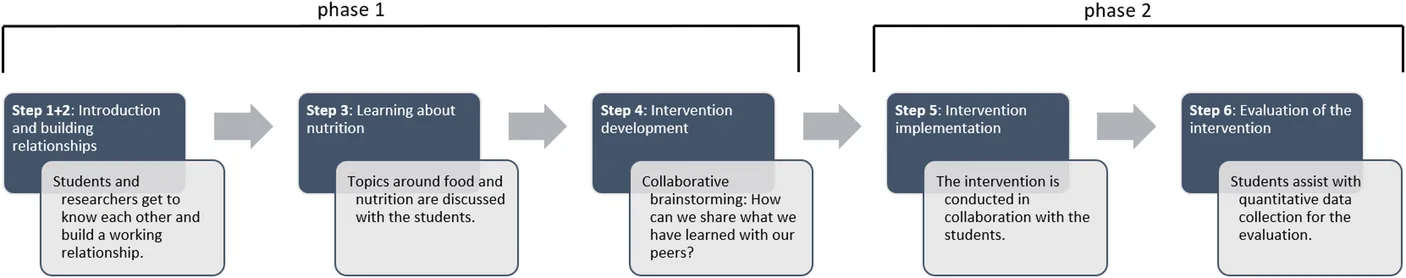
Project workflow: steps and phases of the intervention development, implementation and evaluation

The participatory research project including a formative pilot study was launched in September 2023 and consisted of two phases and several steps (see Fig. 1). During the first phase, children actively participated in designing and developing an intervention to improve their peers’ dietary behaviour. There are several definitions of participatory research and the active involvement of the target group. In this project, we followed the definition provided by INVOLVE, as described in the background section (‘Research that is conducted with or by members of the public rather than to, about or for the public’). Subsequently, the second phase focused on implementing the developed intervention. A more detailed description and the qualitative evaluation of the active participation during the first phase of the project have been published elsewhere. Briefly, the process required openness and flexibility from all involved, with challenges in managing expectations, while children enjoyed the interactive setting, but noted limited hands-on activities (Jilani et al., 2025). This article focuses on the collaborative process of developing a dietary intervention, as well as the second phase, which involved the implementation and its evaluation.

### Setting and Participants

Due to practical and logistical reasons the project could only be implemented at a single site, limiting participation to students enrolled at that school located in a diverse neighbourhood in Bremen, Germany. The neighbourhood is characterized by a predominantly medium to low socioeconomic status, coupled with a high number of residents with a migrant background.

The project was carried out with a group of 6th -grade students (11–12 years old), as the school was only able to accommodate the project within this grade level, limiting participation to this age group. The intervention itself was conducted in 5th -grade students (10–11 years old). A total of 90 students visited the 5th grade at the school, and all students had access to the intervention components. Of these, 51 students completed the food neophobia questionnaire both before and after the intervention and had parental consent for participation. The low participation rate was mainly due to student absences on one of the assessment days and missing parental consent. Teachers reported that failure to return signed consent forms is a common issue at the school. The institutional review board of the University of Bremen gave ethical approval. Parents or legal guardians provided informed consent for children; children provided assent. Parent consent forms were provided in class for children to give to their parents or legal guardians.

### Procedures and Students’ Involvement

Two nutritional scientists (HJ and UK) engaged with a group of 6th -grade students (11–12 years old) in an activity group called “nutrition” during the school year 2023/24. As part of their curriculum, students were required to choose from five different after-school activity groups, four of which offered sports activities, with the fifth being the “nutrition” group. Although attendance of an activity group was mandatory, participation in the “nutrition” group meetings was voluntary to foster an open environment. While 15 students (12 girls, 3 boys) were enrolled in the weekly group, approximately eight (6 girls, 2 boys) regularly attended.

The “nutrition” activity group met once a week for 90 min over the course of 10 months. Meetings during first term took place in a regular classroom at the school, and meetings during the second term were held in the school cafeteria which was not in use during that time. The school cafeteria was located in a central part of the school and had basic food preparation and storage facilities, catering facilities, and seating.

The overarching aim of the meetings during the first school term was to develop an intervention with the active participation of the students. Steps 1–4 were previously described in detail (Jilani et al., 2025). This first phase focused on the development of the intervention. During the second phase, which coincided with the second term, the intervention was implemented by the 6th -grade students of the activity group, who also assisted with the assessment of food neophobia. Figure 1 illustrates the workflow of the project along its different phases and steps. Overall, the steps were interconnected and flowed seamlessly into one another.

### Steps 1 and 2: Introduction and Building Relationships

During the first 8 to 10 weeks of group meetings, the primary focus was on establishing a working relationship with the students, which constituted a prerequisite for meaningful participation in later project phases. In the initial meeting, the students were informed about the overall aims of group meetings and the participatory approach of the project. The intention was to form an active collaboration between students and researchers as equals, allowing the students to contribute as experts of their own nutrition. The following meetings focused on getting to know each other, understanding expectations, and assessing students’ motivation for participation. These aims were supported through small interactive activities and open discussions about their favourite foods and any topics that interested the students. This phase primarily served a formative function by building trust, establishing shared goals, and creating a foundation for sustained engagement in the participatory process.

### Step 3: Learning About Nutrition

The third step aimed to expand students’ knowledge of food and nutrition while maintaining a non-normative and inclusive learning environment. Discussions covering different food groups were introduced, deliberately avoiding the term “healthy” during the first three months of the project. Instead, conversations focused broadly on eating and drinking to encourage open dialogue and reduce moralizing or prescriptive framing.

An external nutrition expert was invited to provide an age-appropriate introduction to healthy eating. In addition, researchers regularly brought food to the meetings to expose students to a wide variety of foods, including fruits and vegetables, whole-grain bread, nuts and seeds, yoghurt, and vegan bread spreads. This phase combined knowledge acquisition with experiential learning and supported students’ preparedness for later involvement in intervention development.

### Step 4: Intervention Development

Building on the groundwork established in Steps 1–3, the focus shifted to the collaborative development of the intervention and its components. The primary aim of this phase was to enable students to transfer their newly acquired knowledge to peers and to actively shape an intervention addressing children’s dietary behaviours.

The only predefined requirement was a focus on children’s diet, while specific components, settings, and implementation strategies remained open. During a collaborative goal-setting session, students generated intervention ideas based on their interests and preferences. The school was identified as a suitable and accessible setting. A strong and consistent student preference emerged for preparing and offering food themselves, which remained central even after alternative options were explored. Consequently, the intervention focused on the provision of healthy snacks within the school environment. In addition, poster development was identified during the brainstorming session as a feasible and effective complementary strategy, and students subsequently designed posters featuring simple, age-appropriate healthy eating messages.

In line with participatory research principles, researchers actively supported the development process while ensuring feasibility within local school conditions. Students were informed about practical constraints and involved in all subsequent planning stages. Their contributions included surveying peers about cafeteria food preferences, gathering information on cafeteria opening hours, experimenting with research methods, collecting ideas for suitable healthy snack options, and designing posters conveying simple healthy eating messages.

Logistical considerations such as time, space, and food preparation facilities further shaped the intervention design. Given the limited duration of group meetings (90 min per week) and restricted kitchen facilities, only small portions of snacks could be distributed to a defined group. By mutual agreement, 5th -grade students were selected as the intervention’s target group. Snack selection was conducted collaboratively through brainstorming sessions and adapted to available resources (Table 1).

Due to scheduling constraints (school holidays, field trips, other obligations by the researchers), participating 6th graders were able to distribute healthy snacks to their peers at five points in April and May 2024.

**Table 1:** Description of provided snacks

	Snacks
1	Whole-grain curd rolls with tomato-sunflower seed spread
2	Pumpkin muffins
3	Homemade pizza with bell peppers and whole-grain dough
4	Yogurt with chia seeds and pureed mango
5	Lentil bread with herbal cream cheese

Overall, the intervention emerged from the integration of the predefined, underlying study aim (to improve dietary behaviours among children) with the components prioritized by the students, namely free food distribution and informational posters implemented within the school setting. Therefore, intervention components were context-specific and emergent (Table 2).

**Table 2:** Intervention design and results of the participatory process of 6th -grade students of the activity group

	Resulting from the active participation (co-design) of 6th graders
Target group	5th -grade students
Setting	School (hallway during long lesson break)
Aims	Introducing new healthy snacks
Content	Healthy eating and food variety
Components	• Free distribution of healthy and self-made snacks which may contain unknown/new foods • Display of posters designed by the 6th graders on healthy eating
Duration	Food distribution at five occasions in May and June 2024

### Step 5: Intervention Implementation

In Phase 2 of the project, students transitioned from co-designers to active implementers of the intervention. The primary aim of this phase was to strengthen student engagement and empowerment by positioning 6th -grade participants as peer educators, thereby putting key participatory principles such as shared ownership, responsibility, and peer-to-peer knowledge transfer into practice.

Healthy snacks were prepared during scheduled activity group meetings, stored at school, and distributed the following day to 5th -grade students during a 30-minute lesson break. Activity group members organized the distribution process by setting up a food station near the classrooms. During the break, peers could freely collect snacks until supplies were exhausted. Group members actively informed peers about the food, explained key ingredients, and used posters to reinforce simple healthy eating messages. After distribution, students cleaned the stations and utensils, further emphasizing ownership of the process. A researcher was present to provide support while maintaining a primarily supervisory role.

### Step 6: Evaluation of the Intervention

The final step explored whether an intervention co-designed with the active participation of children had any observable impact on peers or other stakeholders. Given the formative nature of the project, outcomes were exploratory. Due to practical constraints within the school setting, no control group could be included. Consequently, the evaluation followed an exploratory, pre–post design without a comparison group. Both quantitative and qualitative methods were employed. Quantitative evaluation was conducted using a child-friendly FNS (see below). Activity group members assisted with the initial and final administration of the questionnaire to 5th -grade students, supporting classroom distribution and thereby participating in multiple stages of the research process. Students were not involved in data analysis, the narrative qualitative evaluation or the preparation of this paper, which remained the responsibility of the research team.

## Evaluation

### Narrative Evaluation

The qualitative evaluation of the research project involved a narrative account of observations made by the researchers during the project and intervention implementation. Researchers (HJ and UK) kept detailed minutes and post-meeting notes throughout each phase and step. Both independently analyzed their notes, documenting notable changes or specific characteristics observed in the participating 6th -grade students. Additionally, informal feedback from students, teachers, and other school staff was gathered to supplement these observations. Subsequently, UK compiled a synopsis, which was reviewed and discussed with HJ.

Unfortunately, due to limitations of the ethical approval and the data protection plan, it was not foreseen to formally interview or survey participating students and teachers. Consequently, no formal assessments could be conducted to assess experiences in 6th graders’ with regard to confidence, self-esteem, problem-solving skills or their perceptions of the project. Similarly, feedback on the food offerings could not be collected from the 5th graders, limiting insights into their experiences and preferences.

### Exploratory Evaluation: Food Neophobia

Food neophobia was assessed as an exploratory outcome to examine potential changes in 5th -grade students food-related behaviours following the intervention. The questionnaire was originally developed for adults by Hobden and Pliner (1992) and adapted for the use in children by Laureati et al. (2015). It has previously been described as an effective instrument for assessing the effects of interventions aimed at changing food behaviours in children (Proserpio et al., 2020). Food neophobia refers to the reluctance to eat unknown foods (Pliner & Hobden, 1992) and has been found to affect children’s dietary choices and quality (Kral, 2018; Proserpio et al., 2020). Given the formative nature of the project, the FNS was not used to test intervention effectiveness but to explore potential trends and responses among participating 5th -grade students although it is acknowledged that food neophobia is generally considered as a relatively stable trait.

**Figure 2:**
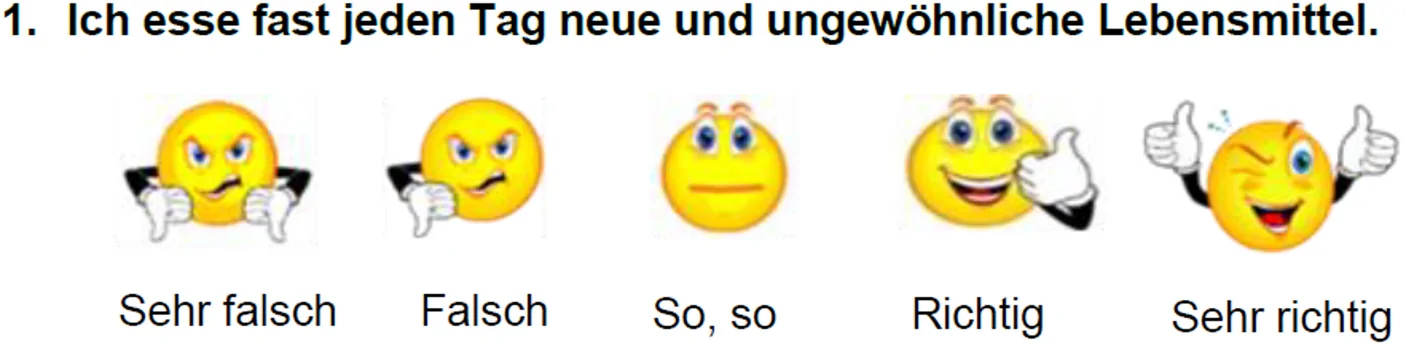
Excerpt from the German version of the Italian child food neophobia scale (Laureati et al., 2015). Translation to english: “I eat new and unusual food almost every day.“; from left to right: “Very false”, “False”, “So, so”, “True”, “Very true”

The child-friendly questionnaire comprises eight items that depict four neophobic and four neophilic food situations. For each item, children responded using a 5-point scale with facial expressions (emojis) indicating varying levels of agreement. The scale ranged from “very false for me” (a frowning face with thumbs down), “false for me” (a frowning face with one thumb down), “so-so” (a neutral face), “true for me” (a smiling face with one thumb up), to “very true for me” (a smiling face with both thumbs up). Emojis, familiar to children, help frame the research task as a game-like activity, enhancing their motivation and focus. Additionally, as a non-verbal method, emojis provide a standardized, universal approach to measuring children’s food behaviour across countries (Gallo et al., 2017). Figure 2 shows an excerpt from the German version of the Italian Child Food Neophobia Scale.

Fifth-grade students completed the FNS one week before the start of the intervention (baseline, April 2024) and two weeks after the end of the intervention (post-intervention, June 2024). Students independently filled out the questionnaire at their school, with a teacher and at least one researcher present to assist students should questions arise. All students who completed the questionnaire had access to all components of the intervention.

Additionally, to check the reliability and temporal stability of the FNS, a subset of children was re-tested one week after baseline assessment. In total, 23 students filled out the questionnaire at baseline and for re-testing and had parental consent for participation. They completed the FNS following the same procedures as in the initial test. This time interval was chosen as it is long enough to avoid recall but short enough to assume that investigated measures have not changed.

#### Data Analysis

The responses to the eight items of the FNS were totalled (with items 1, 4, 5, and 8 being reverse-scored) to obtain an FNS score ranging from 8 to 40. A higher score reflects a greater level of food neophobia. The frequency distribution of food neophobia scores was calculated overall and by gender. Children were divided into three groups based on the 25th and 75th percentiles calculated from the entire sample at baseline: “low food neophobia” (children in the lowest quartile with scores ≤ 19), “medium food neophobia” (children in the mid 50% with scores between 20 and 23), and “high food neophobia” (children in the highest quartile with scores ≥ 24). Reliability of the tool was assessed by calculating internal consistency (Cronbach’s α) and temporal stability by test-retest evaluation (*N* = 23). Analysis of Cronbach’s α was performed with data from the baseline and re-test survey. Total internal consistency was 0.64 and 0.85, respectively, comparable to the suggested value of 0.70 given by Nunnally and Bernstein (1988). For temporal stability, Pearson’s correlation was performed for total FNS scores (*r* = 0.853, *p* = < 0.001). According to QQ-Plots and Shapiro Wilk test, normal distribution was present. To assess for statistically significant differences between baseline and post-intervention FNS scores, we performed paired samples t-test analysis overall and by sex. Statistical analysis was performed in R (version 4.3.2).

## Results

The following section will present the qualitative findings gained from the feedback of involved students and teachers, as well as from the researchers’ observations, along with the results of the FNS, used as a tool for exploratory evaluation of this formative pilot study.

### Observations and Narrative Evaluation

#### Acceptance and Feasibility Within the School Context

Overall, the project was well received by teachers and school administration. Teachers highlighted the value of introducing hands-on activities at the school and noted how much students seem to appreciate the free self-made food (reported by teachers of 5th graders) and the diverse activities in the “nutrition” group (reported by teachers of 6th graders). Teachers further noted that the project contributed to the promotion of practical skills, healthy habits, and a strengthened sense of community within the school, supporting the feasibility of implementing such participatory interventions in this context. However, considerate communication with teachers was necessary when 6th -grade students’ participation was required for the various activities of the “nutrition” group and they needed to leave class early to set up their food station or when food neophobia questionnaires were handed out in class.

#### Student Engagement and Response to the Intervention

The intervention, particularly the free distribution of snacks, was well-received by 5th -grade students. Most students showed high levels of enthusiasm about the free snacks, often returning for second or even third servings. While some students required reminders to collect snacks and a small number initially showed hesitation in approaching the distribution station, students generally reported finding the snacks enjoyable. These observations suggest a high level of acceptability of the intervention components among the target group.

#### Development of Engagement and Group Cohesion Among 6th -Grade Students

While some of the 6th -grade students needed repeated encouragement to actively participate in group activities during the first phase, and several opted to join the more active sports activity groups after the first term, a core group of students emerged that showed a high level of enthusiasm for the activity group meetings and its activities. Jilani et al. (2025) already highlighted how students generally enjoyed attending the group during the project’s first phase. Students that decided to stay on for the second term were very excited and enthusiastic about the group, and also recruited two more equally excited students to the group. Students frequently expressed their affection for the group, often referring to it as “AG-Liebe” (translated as “love for the activity group”) and, towards the end of the school year, expressed sadness that it was soon to be over. This strong group cohesion was further reflected in students actively recruiting additional peers to join the group.

#### Empowerment and Skill Development Through Participation

Active involvement in the development and implementation appeared to foster perceived empowerment among participating 6th -grade students. Indicators of increasing empowerment included students’ growing willingness to express preferences, take responsibility for tasks, and actively shape intervention components, as well as their explicit statements that their opinions mattered within the project. The collaborative working relationship with the reseachers further contributed to a sense of shared ownership.

Over the course of the project, students demonstrated increased confidence, particularly in food preparation and snack distribution activities. Initially, many students showed hesitation and limited experience in handling food and kitchen tools; however their skills and confidence improved noticeably over time. In parallel, students’ interest in food, nutrition, and cooking increased, with several reporting that they recreated recipes at home. These observations suggest that participatory involvement supported both personal skill development and confidence-building among the participating 6th -graders. In addition, their continued enthusiasm was a central process finding and appeared to be a key driver of continued engagement and skill development.

### Exploratory Outcome: Food Neophobia

Fifth-grade students, which were the target group of the intervention, filled out the FNS at baseline (T_0_) and post-intervention (T_1_). Overall, their scores were generally in the mid-range, with boys exhibiting minimally higher levels of food neophobia (Table 3). Table 4 shows the distribution of low, medium and high FNS scores at baseline and post-intervention.

**Table 3:** Food neophobia scores (SD) at baseline and post-intervention, total and by sex

	*N*	FNS T_0_ mean (*SD*)	FNS T_1_ mean (*SD*)
Total	51	22.00 (4.05)	22.43 (4.41)
Girls	25	21.24 (2.93)	21.48 (3.03)
Boys	26	22.73 (4.84)	23.35 (5.32)

*SD* standard deviation; *FNS* food neophobia scale (range 8–40, higher score means higher food neophobia)

**Table 4:** Distribution of food neophobia within the cohort at baseline and post-intervention

	FNS T_0_	FNS T_1_
	*N*	*N*
Low food neophobia (ICFNS ≤ 19)	13	10
Medium food neophobia (ICFNS > 19 & < 24)	24	25
High food neophobia (ICFNS ≥ 24)	14	16

*FNS* food neophobia scale (range 8-40, higher score means higher food neophobia)

As part of the exploratory evaluation, paired sample t-tests were used to descriptively examine FNS scores before and after the intervention. For the overall cohort, there was no significant difference in scores from baseline to post-intervention (*t*(50) = −0.773, *p* = 0.440). No significant difference in scores was observed from baseline to post-intervention, for either girls (*t*(24) = −0.352, *p* = 0.728) or boys (*t*(25) = −0.699, *p* = 0.491).

## Discussion

This formative pilot study aimed to explore the feasibility, acceptability, and engagement potential of a participatory, school-based dietary intervention involving preadolescent students. A central focus was the active involvement of 6th -grade students in the co-design and implementation. Building on their enthusiasm for sharing self-prepared food, 6th -grade students helped design an intervention centred on the distribution of healthy snacks, selected based on their nutrition knowledge and brainstorming during group meetings. The intervention was well received by both students and teachers, and although no significant changes were observed in food neophobia scores among 5th - graders, the project provided meaningful opportunities for 6th -graders to develop confidence, practical skills, and a sense of responsibility in their role as peer educators.

As a formative pilot study, the project was not designed to assess behavioural effectiveness, which would require a different study design. Additionally, the intervention components were context-specific and emergent, which limits generalizability. Instead, the findings highlight the feasibility and acceptability of actively involving preadolescent students in the co-development and implementation of school-based health initiatives.

### Feasibility, Acceptability, and Implementation

Unsurprisingly, 5th -grade students readily welcomed the free food that was provided. Since the school only offers a vending machine with healthier options to this group of students, the free food was naturally well received.

The school administration and teachers clearly valued the hands-on approach of the “nutrition” group and were generally supportive of its activities. However, accommodating these activities was sometimes challenging when they slightly interfered with regular classroom routines, for example, when 6th -grade students needed to leave class early or when the food neophobia questionnaire was distributed during class time. For projects of this kind, the support of school administration, staff and teachers is essential, as external researchers depend on their cooperation to successfully carry out research projects within the school environment and to foster students’ interest in participating. Sustaining the intervention depends largely on the support of teachers. Correspondingly, adult support within a community has been identified as a key factor in the success of participatory projects (Taines, 2012; Torres-Harding et al., 2018). Nonetheless, despite minor disruptions to students’ attendance to lessons, the project could be carried out successfully.

The setting facilitated strong teamwork between 6th -grade students and researchers, as weekly meetings throughout the school year allowed for the establishment of close relationships with the students. The adopted approach allowed for a high degree of flexibility in both the setting and content of the intervention, as no predefined parameters were set. While the school’s facilities and the limited time available restricted what could ultimately be implemented, students’ wishes for a dietary intervention were feasible within the local conditions. Accordingly, little to no compromises had to be made regarding the design of the intervention and its implementation.

### Process and Engagement of 6th -Grade Students

According to various participation typologies for youth participation and empowerment (Wong et al., 2010), this project is characterized by children having both power and responsibility in decision-making. While the project was initiated by adults, the intervention components and its implementation were directed by children, meaning they were actively involved in every step of the planning and implementation process. However, final decisions were made by the adults. Active participation was facilitated by forming a relationship on equal terms between the students and researchers. Students’ perspectives and wishes were taken seriously and had a significant influence on the direction of the project. Collaboration on equal terms has been considered optimal for healthy development (Wong et al., 2010), as young people likely feel valued, respected, and empowered to actively contribute to the decision-making and implementation processes, leading to positive changes in their self-confidence and problem-solving skills (Shah et al., 2023; Torres-Harding et al., 2018; Wong et al., 2010). Indeed, researchers observed meaningful changes in participating 6th -graders as they developed self-esteem and confidence in making decisions related to the project and in handling food. Researchers providing consistent guidance and making the final decisions may have contributed to these meaningful changes, as shared youth-adult control in participatory research has been described as more beneficial for youth development and empowerment than youth-driven participation alone (Wong et al., 2010). Receiving valuable support, feedback and recognition from adults, who may be perceived as role models, can enhance confidence and self-esteem (Kellett, 2010; Wong et al., 2010). This, in turn, may have lasting positive effects, as increased confidence can lead to greater involvement in other aspects of young people’s lives (Kellett, 2010).

Findings demonstrate that school-aged children can successfully and meaningfully participate in school-based dietary health research interventions. Their active participation resulted in significant changes, consistent with findings from similar initiatives. Although not health-focused, Torres-Harding et al. (2018) reported that students participating in a social activism project expressed a high level of enthusiasm, which we also observed within our group. This enthusiasm indicated active engagement and underscores its essential role in producing meaningful outcomes. Additionally, the students’ participation fostered an improvement in confidence, self-esteem, and collaboration skills, which collectively enhanced their empowerment. Similar observations have been made by other research groups (Anselma et al., 2020; Kellett et al., 2004; Shah et al., 2023; Torres-Harding et al., 2018), further highlighting the broad benefits of collaborative youth-led projects. Sixth-grade students also demonstrated an increased interest in nutrition, food, and cooking/baking, and during the second phase they frequently reported recreating recipes at home. Likewise, a Japanese study investigating the effect of a school-based cooking program showed that more children began cooking at home, which further improved their cooking self-efficacy and attitudes towards cooking (Yoshii et al., 2021). This indicates that short cooking programs can improve children’s cooking attitudes, which may be beneficial for their dietary behaviours. A recent systematic review also reported on the positive effects of school-based cooking programs (Vaughan et al., 2024).

### Descriptive Findings for 5th -Grade Students

No significant changes in food neophobia scores were observed among 5th -grade students, consistent with the formative pilot nature of the study, which was not designed or powered to detect intervention effect. Rather, these findings provide descriptive insight into baseline levels and short-term patterns of food neophobia within the target group and help inform the design, exposure frequency, and evaluation strategies of future, larger-scale studies.

By calculating internal consistency and performing test-retest evaluation we were able to confirm the FNS questionnaire’s reliability and temporal stability. However, it should be noted as a limitation that the internal validity across the entire sample was slightly below the commonly accepted threshold of 0.70. Additionally, several factors may explain the lack of significant results for food neophobia. First, the intervention was relatively short, and 5th -graders only received food on five occasions, which may not have been enough to produce significant changes in food neophobia or their willingness to taste unknown foods. Second, due to the large number of students, it was not possible to ensure that every student was able or willing to pick up their portion. As a result, some students may not have consistently received their food, potentially reducing the overall impact on their food neophobia scores. Lastly, food neophobia is highest among children between the ages of 1 and 6, after which it decreases until early adulthood, where it stabilizes (Hazley et al., 2022). Consequently, 5th -graders may already have relatively stable dietary habits and preferences.

## Study Implications and Conclusion

Our findings suggest that participatory, school-based interventions involving preadolescent students are feasible and acceptable within the constraints of everyday school life. Even with limited facilities, time, and organizational flexibility, meaningful activities can be co-designed and implemented when students are actively involved in the process. This highlights the potential of schools as practical environments for collaborative, student-centred projects that extend beyond traditional classroom instruction. Furthermore, the study underscores the importance of support from the school administration, teachers and parents for successful implementation, which requires considerate and clear communication.

Additionally, the active involvement of 6th -grade students in planning and implementing the intervention demonstrated the developmental value of participatory approaches. While our evaluation would have benefited from a formal assessment of these concepts, their engagement increased levels of confidence, responsibility, teamwork, and practical skills, suggesting that such projects may support broader educational and personal development outcomes beyond the immediate topic area. Correspondingly, the inclusion of structured opportunities for joint decision-making between students and adults has been cited as a promising strategy for promoting empowerment and sustained engagement (Wong et al., 2010).

Overall, these findings highlight the value of engaging students as active partners in school-based initiatives and provide practical guidance for designing participatory projects that foster both learning and personal development. Future projects to explore active participation in health research may benefit from involving a wider range of age-groups and more flexible local conditions, including time and facilities.
